# Human Gut Microbiota: Toward an Ecology of Disease

**DOI:** 10.3389/fmicb.2017.01265

**Published:** 2017-07-17

**Authors:** Susannah Selber-Hnatiw, Belise Rukundo, Masoumeh Ahmadi, Hayfa Akoubi, Hend Al-Bizri, Adelekan F. Aliu, Tanyi U. Ambeaghen, Lilit Avetisyan, Irmak Bahar, Alexandra Baird, Fatema Begum, Hélène Ben Soussan, Virginie Blondeau-Éthier, Roxane Bordaries, Helene Bramwell, Alicia Briggs, Richard Bui, Matthew Carnevale, Marisa Chancharoen, Talia Chevassus, Jin H. Choi, Karyne Coulombe, Florence Couvrette, Samantha D'Abreau, Meghan Davies, Marie-Pier Desbiens, Tamara Di Maulo, Sean-Anthony Di Paolo, Sabrina Do Ponte, Priscyla dos Santos Ribeiro, Laure-Anne Dubuc-Kanary, Paola K. Duncan, Frédérique Dupuis, Sara El-Nounou, Christina N. Eyangos, Natasha K. Ferguson, Nancy R. Flores-Chinchilla, Tanya Fotakis, Mariam Gado Oumarou H D, Metodi Georgiev, Seyedehnazanin Ghiassy, Natalija Glibetic, Julien Grégoire Bouchard, Tazkia Hassan, Iman Huseen, Marlon-Francis Ibuna Quilatan, Tania Iozzo, Safina Islam, Dilan B. Jaunky, Aniththa Jeyasegaram, Marc-André Johnston, Matthew R. Kahler, Kiranpreet Kaler, Cedric Kamani, Hessam Karimian Rad, Elisavet Konidis, Filip Konieczny, Sandra Kurianowicz, Philippe Lamothe, Karina Legros, Sebastien Leroux, Jun Li, Monica E. Lozano Rodriguez, Sean Luponio-Yoffe, Yara Maalouf, Jessica Mantha, Melissa McCormick, Pamela Mondragon, Thivaedee Narayana, Elizaveta Neretin, Thi T. T. Nguyen, Ian Niu, Romeo B. Nkemazem, Martin O'Donovan, Matthew Oueis, Stevens Paquette, Nehal Patel, Emily Pecsi, Jackie Peters, Annie Pettorelli, Cassandra Poirier, Victoria R. Pompa, Harshvardhan Rajen, Reginald-Olivier Ralph, Josué Rosales-Vasquez, Daria Rubinshtein, Surya Sakr, Mohammad S. Sebai, Lisa Serravalle, Fily Sidibe, Ahnjana Sinnathurai, Dominique Soho, Adithi Sundarakrishnan, Veronika Svistkova, Tsolaye E. Ugbeye, Megan S. Vasconcelos, Michael Vincelli, Olga Voitovich, Pamela Vrabel, Lu Wang, Maryse Wasfi, Cong Y. Zha, Chiara Gamberi

**Affiliations:** Department of Biology, Concordia University Montréal, QC, Canada

**Keywords:** human gut microbiota, host-microbe interactions, dysbiosis, disease, gut ecology

## Abstract

Composed of trillions of individual microbes, the human gut microbiota has adapted to the uniquely diverse environments found in the human intestine. Quickly responding to the variances in the ingested food, the microbiota interacts with the host via reciprocal biochemical signaling to coordinate the exchange of nutrients and proper immune function. Host and microbiota function as a unit which guards its balance against invasion by potential pathogens and which undergoes natural selection. Disturbance of the microbiota composition, or dysbiosis, is often associated with human disease, indicating that, while there seems to be no unique optimal composition of the gut microbiota, a balanced community is crucial for human health. Emerging knowledge of the ecology of the microbiota-host synergy will have an impact on how we implement antibiotic treatment in therapeutics and prophylaxis and how we will consider alternative strategies of global remodeling of the microbiota such as fecal transplants. Here we examine the microbiota-human host relationship from the perspective of the microbial community dynamics.

## Foreword

This review is the result of a pedagogic initiative undertaken during a third year Microbiology course at Concordia University in Montréal. The project's aim was to study the web of interactions between the human host and its gut microbiota by researching the latest discoveries and present it from the perspective of the microorganisms. The initiative also aimed at demonstrating to the students how to research the primary literature and write an analytical, comprehensive and balanced review. Choices on which original sources were cited were exclusively dictated by the pedagogic scope and reach of the course and in no way reflect considerations on the value of the contributions that were excluded. We therefore apologize to all the colleagues whose important research could not be cited due to such constraints.

## Composition of the human gut microbiota

The community of microorganisms residing in the human gastrointestinal (GI) tract, the GI microbiota, is composed of about one thousand commensal, and/or symbiotic microbial species among which Viruses (including bacteriophages), Bacteria, Archaea, and unicellular eukaryotes, comprising Fungi and other non-bacterial and non-archaeal microbial species (Ley et al., 2008; Gerritsen et al., 2011; Lozupone et al., 2012; Keeney et al., 2014; Lagier et al., 2016). Difficulties in culturing some gut microorganisms and discrepancies between the relative species relationships observed *in vivo* and *ex vivo* has resulted in an initial underestimate of the number of species in the human gut microbiome, the list of microbial gene functions of the GI tract (Lagier et al., 2015). Technological advances enabling metagenomics and microbial identification via MALDI-TOF mass spectrometry and the more recent implementation of culturomics have expanded the known microbial community of the human microbiome (Gill et al., 2006; Ley et al., 2006; Seng et al., 2013; Hugon et al., 2015; Lagier et al., 2016). Under normal healthy circumstances GI tract microbes can perform beneficial tasks for the human host, e.g., food break down, synthesis of vitamins and biomolecules and interaction with its immune system. The GI environment, in turn, may support the growth, reproduction and longevity of the bacterial community (Lozupone et al., 2012; Browne et al., 2016). Changes in the gut environment from diet and host physiology, as well as ingested microbes, may create competition for resources that affect the resident microbiota and may re-shape the microbial community, which in turn may affect host physiology. Host energy, metabolism and immunity have been found to respond to cues from the GI microbiota and many human health conditions have been linked with particular compositions of the GI microbiome (Bäckhed et al., 2005; Robosky, 2005; Turnbaugh et al., 2006; Rohde et al., 2007; Hibbing et al., 2010; Sommer and Bäckhed, 2013).

The gut-associated microbiome was found to be relatively conserved among several vertebrates including human, mouse, and zebrafish and it was speculated that ancestral mammalian species as early as the Jurassic period, relied on their gut-microbes to support their mainly herbivorous diet (MacKie, 2002; Rawls et al., 2004; Ley et al., 2008). It has been proposed that throughout mammalian evolution carbohydrates became prominent energy sources and the acquisition of gut-microbes may have enabled a large fermentative platform to supply the host with essential biomolecules (MacKie, 2002; Gill et al., 2006; Ley et al., 2008; Conlon and Bird, 2014). Constantly shaped by its interaction with the host, the human GI microbiota plausibly diverged from other microbial communities found in nature (Thaiss et al., 2016a). Consistent with this possibility, culture-free genetic profiling of the 16S ribosomal (r) RNAs revealed major differences between free-living, or non-animal-associated, microbial communities and gut-associated microbiomes (Ley et al., 2008; Lagier et al., 2016). The microbiota and the host co-evolve with each other, albeit at different speed. Experiments in murine models provide growing evidence of deep relationships between the GI microbiota and the host physiology and gene expression (e.g., Stappenbeck et al., 2002; Round and Mazmanian, 2009; Dalmasso et al., 2011; Ghosh et al., 2011; Larsson et al., 2012; Reinhardt et al., 2012; Thaiss et al., 2016a). The adult GI microbiota was found to have a large inter-individual variation, with over 1,000 different bacterial species (Qin et al., 2010; Guinane et al., 2013). Archaea (especially the genus *Methanobrevibacter*) and fungi (phyla Ascomycota and Basidiomycota) also characterize a large portion of the human GI microbiome (Hoffmann et al., 2013). 16S rRNA analyses have revealed that more than 90% of bacterial species found within the gut are Bacteroidetes and Firmicutes, as well as Proteobacteria (Hold et al., 2002; Wang et al., 2003; Qin et al., 2010; Guinane et al., 2013). Proteobacteria and Bacteroidetes are Gram-negative bacteria involved in carbohydrate digestion, gut microbiome development, immune system modulation, and protection against colonization by pathogens (Russell et al., 2014). Some Firmicutes are Gram-positive bacteria that also digest carbohydrates (Berry, 2016). Human-associated Archaea, among which *Methanobrevibacter smithii* found in almost 96% of healthy subjects, synthesize methane from H_2_ produced by bacterial catabolism (Hoffmann et al., 2013; Lurie-Weinberger and Gophna, 2015). Human-associated fungal species include *Saccharomyces, Candida*, and *Cladosporium*, and several low-abundance strains (Hoffmann et al., 2013). *M. smithii, Saccharomyces*, and *Candida* were frequently found together in individuals having carbohydrate-rich diets (Hoffmann et al., 2013). While *Candida* remains asymptomatic in many individuals, immuno-suppression and/or antibiotic treatment may promote outgrowth and niche specific invasion throughout host tissues and mucosal sites (Huffnagle and Noverr, 2013). The GI microbiota appears sustained through complex interaction networks between the main phyla with the observed microbial proportions, likely reflecting the range of reciprocal exchanges needed for the dynamic physiological balance of both microbiota and the host (Qin et al., 2010; Cho and Blaser, 2012; Lagkouvardos et al., 2017). The GI microbiota also appeared relatively resilient and stable (Faith et al., 2013; Schloissnig et al., 2013) which seemed to suggest the possibility of developing new biomarkers for diagnostics and possibly for therapeutic purpose. Within single individuals, the environment changes along the GI tract, thus different microbes and population densities are found in a continuum along the different regions, reflecting their adaptation to distinct pH, enzymatic conditions, anatomical structures, and physiology (Schneeman, 2002). For example, the pH in the mouth is close to neutral and the saliva contains enzymes inhibiting bacterial growth, while the stomach is extremely acidic (pH 2). The pH gradually increases in the small intestine and the colon (pH 4–5 and 7 respectively, Evans et al., 1988). Analyses of the human microbiome by the Human Microbiome Project revealed that, besides the dominant phyla, *Bacteroidetes* and *Firmicutes*, there was a great variation in relative composition of the microbiota, both in phyla-genus distributions, as well as individual differences that were initially grouped into different enterotypes (HMPC, 2012a,b). In the upper digestive tract stomach and small intestine, dwell abundant aerobic and facultative anaerobic species, while in the lower GI tract reside anaerobic species. In addition, functionally redundant species were found in multiple niches of the GI tract (Sommer and Bäckhed, 2013; Lozupone et al., 2012; Browne et al., 2016). As studies were extended to more and more subjects it became increasingly clear that individual variability was higher than anticipated and there was a need to revise the enterotype concept from one of fixed categories to one of a continuum along multiple dimensions (Knights et al., 2014). Interestingly, re-analyses of a sample time course from a single individual revealed that the GI microbiome may change enterotype, presumably indicating its adaptive capacity (ib.).

That no unique or discrete formula for a “healthy microbiota” seems to exist may reflect microbial functional redundancy enabling multiple species to play similar roles in the microbiota-host relationship. Other factors may also contribute, such as genetics and diet (Turnbaugh et al., 2007; Qin et al., 2010). In the context of the relationship between host and microbiota it seems advantageous that a healthy microbiota be able to carry out certain metabolic functions, such as providing short chain fatty acids (SCFA) to the intestinal epithelial cells. Metagenomic analysis showed that gene functionality of healthy microbiota appears indeed similar across individuals (HMPC, 2012b). Recent metabolomics studies have also revealed diverse inter-individual profiles that promise to yield biomarkers for various diseased states (reviewed in Vernocchi et al., 2016). Relative balance among different microbial groups seems important for the host health and pathological alterations of such balance are defined as dysbioses (Tamboli et al., 2004). Regardless of its cause, dysbiosis correlated with various pathological conditions of the host, such as Inflammatory Bowel Disease (IBD), Type 1 Diabetes, Rheumatoid Arthritis (RA), asthma and obesity (reviewed in Keeney et al., 2014) and there is suggestive evidence that the gut microbiota may also be implicated in cancer (Ray and Kidane, 2016; Wroblewski et al., 2016; Yamamoto and Matsumoto, 2016) and may at least influence certain mental health conditions via the gut-brain axis (Moos et al., 2016; Mu et al., 2016; Obata and Pachnis, 2016).

Because of its enormous surface separating digesting food from the body and its role in nutrient absorption, the intestine is a fundamental barrier for the integrity and health of the organism, which is constantly patrolled by the immune system. In its complex synergy with the host's immune system, the GI microbiota stimulates and is in turn stimulated by innate immunity, a balance that can be altered by pathogens trying to access the riches of the intestinal ecological niche (Thaiss et al., 2016b). During infection innate immunity and the resident microbiota cooperate to displace the pathogens actively via antimicrobial molecules and passively by crowding the niche and impeding the pathogen attachment to the gut lining. Simultaneously, the host-microbiota preferential relationship is promoted by secreted fucosylated molecules on the surface of the intestinal epithelial cells that favor resident microbes (Pickard et al., 2014).

**Figure 1:**
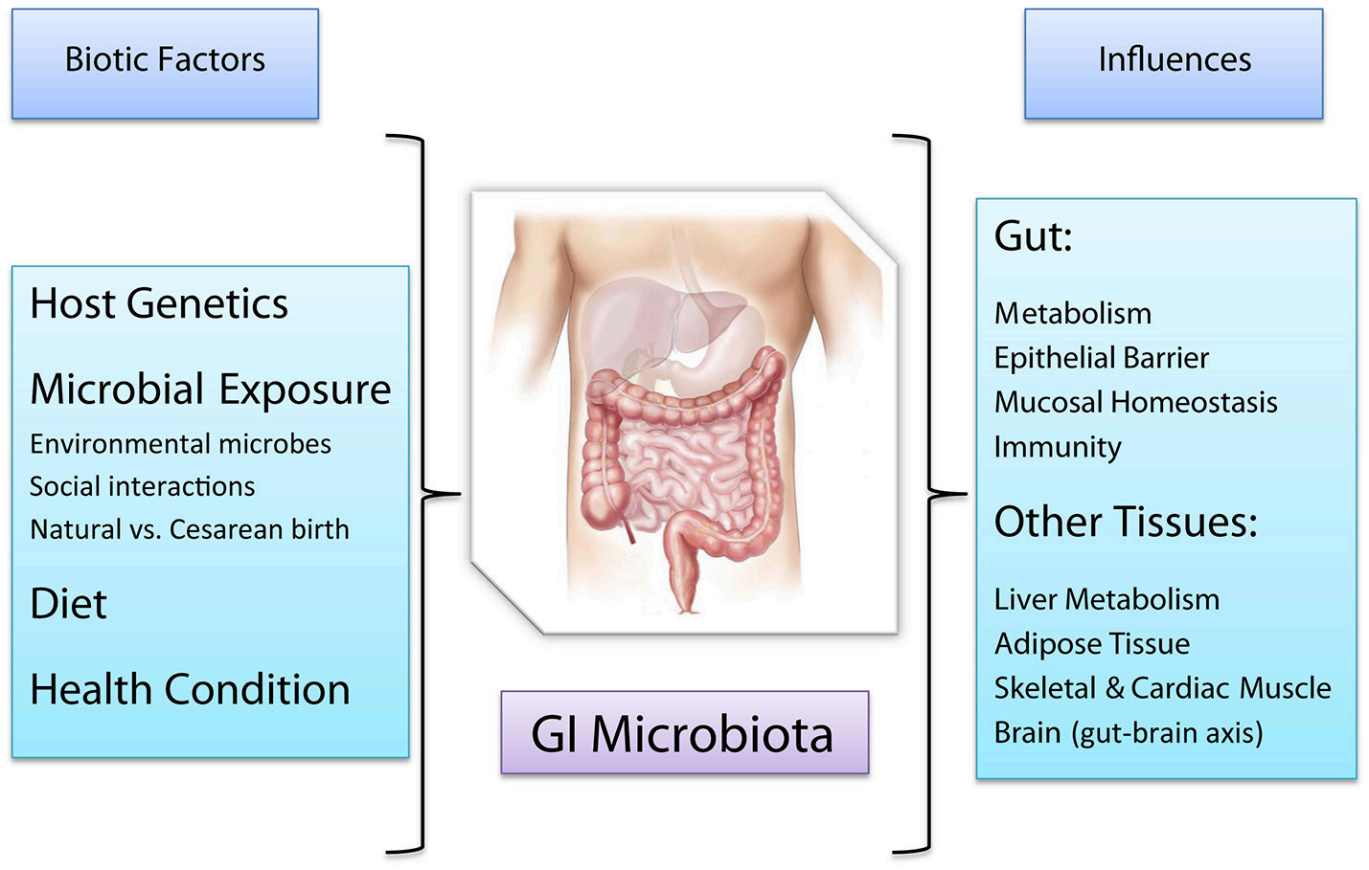
Factors affecting the human GI microbiota and host functions affected, either directly or indirectly, by the GI microbiota.

We now realize the enormous impact the gut microbiota has onto human health and it has become an intensely studied subject, with a wealth of information being produced. This review examines recent discoveries about the diverse relationships between the human host and its microbiota from the perspective of the biological interactions of mutualistic symbiosis and/or commensalism between microorganisms and their human host (Figure 1). As we discover the many nuances of these complex connections and the underlying biomolecular associations, we raise questions about the partners' autonomy in the host-microbiota relationship. The holobiont theory describes the host-microbiota as a single biological unit, called the holobiont, which functions dynamically and appears to be subjected to evolution, rather than—or possibly in addition to—the individual species composing it (Bordenstein and Theis, 2015). Despite their poly-genomic composition, the deeply symbiotic relationships that have persisted among hosts and microbes across many groups and over time was used to define a consortium of interactions suspected to be cohesive with respect to selection processes (Booth, 2014; Bordenstein and Theis, 2015). Still controversial, the concept of the hologenome (i.e., the gene ensemble comprising a host and microbe syndicate) relies on cooperative integrated features of the host-microbiome partners with high fidelity of co-transmission, the latter being essential for being subjected to selective pressure (Zilber-Rosenberg and Rosenberg, 2008; Gilbert et al., 2012; Rosenberg and Zilber-Rosenberg, 2013; Douglas and Werren, 2016). Appealing for its inclusion of the functional relationships between host and microbiota, the holobiome concept is difficult to apply in the case of horizontally acquired symbionts, which do not have cohesive genomes with respect to different individuals (Douglas and Werren, 2016). Rare examples may include the endosymbiotic acquisition of nuclear organelles, which would be, however, an integrated part of a hologenome and not an entire microbiome (Douglas and Werren, 2016). As such, the holobiont and hologenome concepts have demonstrated poor research utility (Douglas and Werren, 2016).

## Functions of the GI microbiota: nutrient processing and availability

The GI microbiota contributes to the host's digestion by making carbohydrates available (Stecher, 2013; Conlon and Bird, 2014). Among the resident bacteria, species belonging to the phyla *Bacteriodetes* and *Firmicutes* can ferment undigestible carbohydrates, the fiber, to produce short chain fatty acids (SCFAs), branched fatty acids, lactate, ethanol, CO_2_, and H_2_ which are then utilized by secondary fermenters, by the host, or excreted (Cummings et al., 1989; Belenguer et al., 2006; Flint et al., 2008; Fischbach and Sonnenburg, 2011; Sheridan et al., 2016; Patrascu et al., 2017). Of note, microbiota-produced SCFA constitute the main energy source of the intestinal epithelial cells (Topping and Clifton, 2001; Louis et al., 2010). Bacteria in the human gut microbiota can also synthesize beneficial vitamins such as folate, biotin, and vitamin K (Cummings and MacFarlane, 1997) and neutralize potentially carcinogenic compounds such as pyrolysates (Morotomi and Mutai, 1986). Gut bacteria detect available polysaccharides and activate different metabolic utilization pathways. In the case of the well-studied *Bacteroidetes* and the *Firmicutes* family *Lachnospiraceae*, these enzymes are encoded by multiple polysaccharide utilization loci (PULs, Bjursell et al., 2006; Martens et al., 2011; Sheridan et al., 2016) conferring the ability to utilize different glycans as carbon sources, which may allow trophic diversification, moderate species competition and facilitate distal gut colonization (Bjursell et al., 2006; Martens et al., 2011; Sheridan et al., 2016). The GI lumen, with its over 200 grams of digested contents, provides a diverse and competitive microbial environment for both mutualistic and pathogenic microorganisms. Successful colonization of a species acquired from the environment may depend on the ability to utilize differential nutrient sources, perform chemical sensing, and coordinate gene expression in favorable ways (Lozupone et al., 2008; Pacheco et al., 2012; Patrascu et al., 2017). Moreover, the presence of “keystone species” with rare metabolic capabilities may generate energy and cross-feed other microbial community members (Ze et al., 2012, 2013). Host genetics, food availability, dietary modifications, and resident microbes will influence contents and environment of the GI tract, conceivably affecting each microbial species differentially, and potentially shaping both species representation and numerical abundance of the community members. These models seem to agree with the results of both observational studies that correlated diet with microbiota composition in individuals from different geographical locations (De Filippo et al., 2010; Yatsuneko et al., 2012; Schnorr et al., 2014) and of human studies employing controlled diets (Walker et al., 2011; Martinez et al., 2013; David et al., 2014; O'Keefe et al., 2015). Because of the impossibility to experimentally control variables such as the individuals' different genetic makeup and individual microbiota composition, the conservative interpretation is that the microbiota was found to respond to dietary changes quickly, with some microbial communities more readily responding to such changes than others (Walker et al., 2011; Salonen et al., 2014). These dynamics are reminiscent of other microbial expansions in natural habitats (e.g., algal blooms) where excess of certain nutrients may favor the over-proliferation of definite groups and outcompete other species in the same environment, reducing overall microbial diversity, and highlighting the potential to adapt ecological models for studying the GI microbiota.

Because of its dynamic nature, the microbiota could potentially be manipulated via dietary changes and administration of either antibiotics or probiotics to, respectively, increase or decrease microbial numbers and diversity. Probiotics can be aliments such as traditional fermented foods rich in live microorganisms (e.g., sauerkraut, kimchi, yogurt) or supplements (Voreades et al., 2014; Zuk, 2014) which have been used to promote proliferation of beneficial microbes and organismal diversity at the expense of selected pathogens in diseases accompanied by dysbiosis (Saxelin et al., 2010). Prebiotics on the other hand, provide molecules to stimulate and support resident GI tract bacteria (Bouhnik et al., 2004, 2006; Ramirez-Farias et al., 2009; Davis et al., 2011; Dewulf et al., 2013). Probiotic administration was found to increase gut microbial diversity in obese patients up to a month (Kadooka et al., 2010; Wassenaar et al., 2014) suggesting the feasibility of directing changes in the microbial imbalance of the GI tract by outcompeting harmful bacteria (Rigottier-Gois et al., 2015). A stimulating prospect, GI microbiota manipulation reminds us that our incomplete knowledge of the GI microbiota ecology and its intricate relationships with the host may result in accidentally favoring opportunistic pathogens or provoking pathological responses (Mukherjee et al., 2013). On the other hand, microbial competition could in principle be employed strategically to limit or eliminate pathogens in alternative to conventional antibiotic treatments, with the added advantage of preventing horizontal transfer of antibiotic resistance genes.

## Immunity and GI microbiota

Early development of the gut microbiota has been shown to be important for normal immune response and to prevent autoimmune disease (Round and Mazmanian, 2009). Because the relationship between host immune system and microbiota has been recently reviewed thoroughly (Belkaid and Harrison, 2017) and most of our co-authors had not yet studied Immunology, a fourth year course, here we will just recall a few concepts for completeness and refer interested readers to Belkaid and Harrison. The GI microbiota is essential for the maturation of the immune system, which is composed by both adaptive and innate immune responses. Innate immunity relies on the physical barrier of the epithelia, circulating chemicals and specialized cells to quickly recognize a broad range of foreign antigens on cells with pathogenic potential and eradicate them (Reece et al., 2011; Lupfer et al., 2014). On the other hand, adaptive immunity deals with pathogens through a slower, albeit long-lasting, response via stimulation of lymphocytes expressing specific cross-reactive antibodies (Lupfer et al., 2014). Displaying the largest density of epithelial cells (EC) in the body, the GI tract receives more antigens than any other body parts through ingested foods and the interactions with the microbiota (Kuhn et al., 2014; Shi et al., 2017). The latter contributes to maintenance of the intestinal barrier at various levels. For example, it stimulates the goblet cells to produce the mucus lining the intestine (Kandori et al., 1996); it is involved in determining the specificity of the luminal immunoglobulins A, which recognize antigens in potential pathogens (Brandtzaeg et al., 2006) and contrasts their adhesion for effective clearance (Johansen et al., 1999; MacPherson and Uhr, 2004). Because every microorganism in the gut, both commensal and foreign, can (and will) be targeted by the host's immune system, it may seem surprising that healthy interactions between the specialized intestinal phagocytic macrophages and the microbiota normally do not cause release of pro-inflammatory cytokines IL-1 and TNF and inflammation (Smythies et al., 2005). How host immunity avoids being overpowered is only partly known and actively being investigated. Because of the high selectivity of the gut environment, only microbes able to establish a “dialog” with the host can colonize the microbiota, while the others will be eliminated by the combined action of the host's immunity and the resident microbes, some of which can synthesize bacteriocins which actively target competing species (Corr et al., 2007; Hibbing et al., 2010; Patwa et al., 2011; Dobson et al., 2012). Interestingly, germ-free mice were found to contain fewer circulating lymphocytes (Wostmann et al., 1970) suggesting that GI microbes may also affect adaptive immunity.

## Nutrients, bacterial competition, and colonization

The mammalian gut environment is fairly protected and has relatively stable temperature, yet it is dynamic and competitive, imposing a strong evolutionary pressure onto the microbes. The resident microbiota is constantly sensing and integrating information on nutrients and microbial content of digested food and drinks, the glycans projecting from the surface of intestinal cells, and the overall health and immunity of the host. The tight interaction web between host and its GI microbiota allows resident microbial communities to monopolize nutrients and adhesion sites and establish a molecular and genetic “dialog” with the host, simultaneously blocking newcomer, potentially pathogenic, microorganisms from accessing the high energy sources they need to replicate and establish themselves in the community (Stecher, 2013). To successfully colonize the intestine, microorganisms must withstand fast changes in nutrients, high osmolarity and stomach acidity, bile salts, protective mucus, and microbial competition. Antigens detected in unfamiliar bacteria cause phagocytosis from the host's cells. Bacteria that can avoid phagocytosis can begin colonization by proliferating initially in small niches. These localized microbial blooms may cause resource competition and transiently alter gut ecology, although the long-term outcome of the inter- and intra- specific bacterial competition allowing the stable colonization of the new species or its demise likely depends on the context of resource availability, interactions with the microbial species in the same trophic level as well as the host's immunity (de Muinck et al., 2013). Large scale bacterial invasions, as may result from the ingestion of contaminated food or water, may severely compromise the resident microbiota, reducing its diversity in favor of the expansion of selected strains.

The host's nutritional status may affect both microbiota diversity and microbial colonization. How well strains can adapt and access nutritional resources can contribute to reproductive success of both resident microbes and foreign invading species. Upon introduction into the mammalian gut, *Escherichia coli* and other Gram-negative bacteria were observed to undergo many rounds of mutation, producing both adaptive and deleterious variants that spread through the population simultaneously in so called “soft sweeps” (Barroso-Batista et al., 2014). Should any mutation provide survival advantage (or possibly by chance in small populations) it could become established and compete with all others for fixation. In a germ-free mouse model that recapitulates and simplifies the complexity of the natural ecological conditions, inoculation of model *E. coli* strain MG 1655 showed the appearance of the first beneficial mutation just 2 days after invasion, which lead to quick adaptation to the murine host. MG1655 carried loss of function mutations in the *gat* and *srl* operons (which encode the biochemical machinery for utilizing galactidol and sorbitol respectively) enabling the mutants to utilize alternate carbon sources in conditions of high competition for nutrients (ib.). Another adaptive mutation in the *arcA* gene critically enabled switching between aerobic and anaerobic respiration depending on oxygen levels (ib.). In this system, the temporal unfolding of the colonization process appeared influenced by strong selection as well as the contributions of multiple small effect mutations in a process that could surprisingly be repeated, which may intriguingly suggest the existence of predictable driving forces (Lourenço et al., 2016). The capacity of utilizing ready-made metabolic intermediates may also be advantageous for microbes. Vitamin B_12_ is a cofactor required for metabolic processes in over 70% of Gram-negative bacteria whose *de novo* synthesis requires 30 steps. Combinations of metabolically economic systems of transmembrane “corrinoid receptors” for absorbing B_12_ itself or its analogs (collectively termed corrinoids) have been found in many species and appeared to increase the cell's fitness, depending on corrinoid availability (Degnan et al., 2014; Sonnenburg and Sonnenburg, 2014).

Besides mutations affecting nutrient uptake and utilization, mutations in genes with regulatory functions may also provide means of fine-tuning adaptation by affecting downstream pathways both additively and independently (Giraud et al., 2008). While phenotypic variants compete for survival and dominance within the same trophic level, both ecological context (e.g., other microbial species in the same trophic level, host genetics) and environmental resource availability may result in different colonization patterns dynamically and over time. *Ex vivo* studies of simplified models of GI microbiota found that, during the early colonization stages, both aerobic and facultative anaerobic *E. coli* strains could thrive until oxygen reserves were exhausted and obligate anaerobes strains took over (de Muinck et al., 2013). However, introduction of other bacterial species was found to change the outcome: *Clostridium perfringens*, naturally present in the gut microbiota, lowered environmental peptone levels, impairing the success of the anaerobes, which were more dependent on peptone than the aerobic strains (ib.). Host immunity appears to contribute to colonization. Considering the microbiota diversity *in vivo*, we expect that the microbial interaction web will be at least as complex as what has been observed in other environmental poly-microbial communities, with the added component of multiple layers of interaction with the host. In the context of this formidable, yet fascinating complexity, studies of murine models may not fully reproduce the species-specific interactions of the human microbiota with the human host (Chung et al., 2012; Du et al., 2015) yet may provide controlled experimental conditions in which genetic homogeneity and germ-free culturing conditions minimize the influence of compounding factors such as inflammation, thus facilitating the study of the molecular mechanisms of interaction between the host and either single species or controlled microbial mixtures (e.g., the simplified human microbiota, Becker et al., 2011; Eun et al., 2014), whose knowledge contributes to our global understanding of the host-microbiota relationship.

## Nutrient niches favoring pathogenic gut colonization

Resource acquisition is crucial for microbes (including pathogens) aiming at colonizing the competitive environment of the mammalian intestine and these species must overcome the inherent colonization resistance of healthy individuals (Van der Waaij et al., 1971; Faust et al., 2015). In healthy individuals competition for both nutrients and attachment sites make pathogens such as *Clostridium difficile* less likely to colonize the gut and become a burden to the host (Guarner and Malagelada, 2003). The enterohaemorrhagic *E. coli* strain O157:H7 found in undercooked meat and contaminated produce is responsible for many bacterial food poisoning cases in developed nations (Loftsdóttir et al., 2017). What makes O157:H57 successful is its ability to regulate gene expression in response to its surroundings. Commensal *E. coli* are found in the mucus layer, whereas O157:H57, along with non-pathogenic *Bacteroides thetaiotaomicron*, are found on the intestinal epithelium (Iversen et al., 2015). Rather than counterproductively competing for resources in the mucus layer, O157:H57 can colonize areas of high fucose availability on the intestinal EC (Pacheco et al., 2012). *B. thetaiotaomicron* cleaves fucose from host produced mucin, which activates the FusKR receptor in O157:H57 and the expression of virulence factors. Upon successful colonization of the intestinal epithelium, O157:H57 metabolizes carbon sources such as galactose, which are not being utilized by commensal *E. coli*.

*Campylobacter jejuni*, typically acquired through ingestion of uncooked poultry, causes a form of human gastroenteritis (Olson et al., 2008). A highly fermentative healthy microbiota generates steady supplies of organic acids such as acetate and lactate that constitute electron and carbon sources for downstream metabolism. *C. jejuni* was found to contain two completely redundant respiratory enzyme systems for growth on L-lactate, underscoring the evolutionary advantage of being able to grow on lactate (Thomas et al., 2011).

One of the best characterized examples of pathogenic bacterial infection is that of the members of the *Salmonella* genus, that utilize an arsenal of resources during their attempts at gut colonization. *Salmonella enterica* serovar T*yphimurium* (hereby referred to as *S. Typhimurium*) is a common Gram-negative microorganism of the *Enterobacteriaceae* family that causes typhoid fever and food poisoning. All Salmonella serotypes are disease causing in humans, but only certain ones are host specific (WHO, 2016). The dominant strain responsible for human pathogenicity is *S. typhi*, while *S. Typhimurium*, also pathogenic, is transmitted from animals to humans either directly or via contact or consumption of undercooked infected meat (WHO, 2016). In the initial phase of invasion, *S. Typhimurium* generates energy via the *hyb* hydrogenase-mediated oxidation of H_2_, a readily available by-product of resident microbe metabolism (Lamichhane-Khadka et al., 2010; Maier et al., 2013, 2014). Because *hyb* mutants displayed impaired growth, *hyb* function appears necessary for invasion. Moreover, H_2_ sequestration by an avirulent strain able to utilize H_2_, *S. Typhimurium*^avir^, could thwart *S. Typhimurium* colonization (Maier et al., 2013). Energetic strategies based on H_2_ utilization are also employed by other pathogens to gain purchase on the resident microbiota, among which *Campylobacter, Shigella*, and *Yersinia* (ib.). When nutrients are scarce, many bacteria become more motile as they forage for survival. Distinctively, nutrient abundance was found to stimulate a bistable response in *S. Typhimurium* in which a proportion of cells activated the expression of flagellar proteins becoming highly motile and continuing host invasion. The other cells, instead, remained non-motile and non-invasive, bypassed the host's inflammatory response and may fuel subsequent colonization bouts (Koirala et al., 2014). To gain advantage on the commensal species of the microbiota, *S. enterica* can release colicin Col1b, a pore-forming toxin targeting the *Enterobacteriaceae* family and especially *E. coli* (Nedialkova et al., 2014). Because toxin synthesis is energetically expensive and Col1b is toxic to *Salmonella* itself, only few cells in the population were found to secrete colicin (ib.). Interestingly, the success of a colicin invasion strategy may depend in part on nutrient availability, because the presence of iron caused conformational changes of the *E. coli* receptor, reducing its sensitivity to Col1b (Pugsley and Reeves, 1976). Finally, *S. enterica* can form biofilms, robust bacterial sheets protected by a self-secreted polysaccharide matrix which can adhere to surfaces. Albeit likely to occur, we do not have experimental evidence of biofilm formation *in vivo*. If confirmed, such capacity may conceivably contribute to colonization (Chelvam et al., 2014). The capacity for *Salmonella* biofilm formation is encoded on several different plasmids, one being pRST98, whose expression is triggered by extracellular signaling molecules N-acylhomoserine lactones, which are part of the quorum sensing system activated by bacteria in crowded populations (Liu et al., 2014).

## Microbiota manipulation of host's behavior?

While commensal GI microorganisms can extract nutrients from the food, they do require specific nutrients to thrive. During studies on the causative factors of obesity it was observed that the GI microbiota may modulate expression of taste receptors (Miras and le Roux, 2013; Avau et al., 2015; Avau and Depoortere, 2015; Murtaza et al., 2017), affect the vagus nerve which controls the gut-brain axis (Rhee et al., 2009; Bercik et al., 2012; Collins et al., 2012; Vaughn et al., 2017) and influence the release of toxins and neuro-transmitters (Kollai et al., 1994; Lyte et al., 2011; Clarke et al., 2014). In a small human study, probiotics were found to stimulate both number and diversity of gut microbiota in obese adults and reduced host cravings, raising the untested possibility that the subjects' eating preferences may have been modified (Kadooka et al., 2010). Initial attempts at probing this possibility in mice made use of either probiotics or antibiotics administration to alter the GI microbiota and observe changes in the hosts' eating patterns and food preferences, which appeared to correlate with the nutritional requirements of the current resident microbes (Alcock et al., 2014; Zuk, 2014). The ratio of *Bacteroidetes* to *Firmicute*s is often used as an indicator for body mass, with high ratio observed in lean individuals and low ratio in obese ones respectively (Bäckhed et al., 2004; Ley et al., 2005; Turnbaugh et al., 2006, 2007; Zhang et al., 2012; Evans et al., 2014; Lecomte et al., 2015; Shang et al., 2017). The *Firmicute* family *Lachnospiraceae* is responsible for producing SCFAs that can fuel the growth of other gut microbes and host epithelial cells, and may enhance the host's energy harvesting capacity, resulting in liver lipogenesis and triglyceride accumulation (Meehan and Beiko, 2014; Singh et al., 2015; Nehra et al., 2016; Shang et al., 2017). Consumption of certain food groups seemed to affect both the *Bacteroidetes* to *Firmicutes* ratio and the diversity in the gut, which in turn may influence the gut-brain vagal communication, potentially contributing to obesity (de la Serre et al., 2015; Sen et al., 2017). These intriguing results suggest that the microbiota may influence the host's eating preferences affecting its mood and satisfaction for certain food groups, possibly depending on the nutritional requirements of the members of the microbial community. Only supported by correlative evidence, this speculative scenario raises interesting points of reflection. Considering that each microorganism in the microbiota could potentially and competitively manipulate the host for its own benefit, a diverse microbiota would favor the host by lessening the intensity of the influence by any single microbial group. Conversely, a microbiota composed of fewer, more numerous groups may potentially exert a stronger influence on host eating behavior (Alcock et al., 2014), which would agree with the peculiar microbiota composition observed in certain eating disorders.

## The microbiota in fasting and malnutrition

Dynamically responding to environmental conditions, the microbiome responds to nutritional changes, including fasting and malnutrition (Arumugam et al., 2011; Flint et al., 2014). Because of ethical reasons the effects of starvation on the GI microbiota has only been studied in controlled settings in animal models. Several weeks of nutrient deprivation in fish, mice and toads displayed increased microbiome diversity; quails showed no change, and geckos a decrease (Kohl et al., 2014). Seasonal fasting can occur naturally, e.g., during molting of king and small penguins, when energy reserves whose deposition depends on gut function become essential for thermoregulation and producing new plumage. Molting penguins exhibited changes in microbiota composition, especially after prolonged fasting. King penguins displayed increased Proteobacteria during early molt, and decrease of both Proteobacteria and Bacteroidetes during late molt (Dewar et al., 2014). Conversely, in hibernating squirrels the normally dominant Firmicutes group contracted throughout hibernation. A small, representative bacterial population seeded the rebound of gut microbiota during the active months (Dill-Mcfarland et al., 2014). These variable responses to starvation make it difficult to model the effects of nutrient deprivation in humans, which we can only observe in specific cases of malnourished individuals. Human *moderate* and *severe acute malnutrition* are significant global health issues. Respectively affecting ~19% of children in Asia and 20 million children globally (WHO, 2007), they are leading causes of child mortality (Smith et al., 2013). Severe malnutrition is typically treated with nutrient-rich “ready-to-use therapeutic food,” which reduces mortality, albeit children cannot fully recover even after body mass improvements, likely because of their immature microbiomes (Prentice et al., 2013; Tilg and Moschen, 2013; Subramanian et al., 2014). In fact, early gut microbiome development is particularly important in children, whose microbiota shifts as they grow and change diet (Bergstrom et al., 2014). Microbiota remodeling by antibiotic therapy improved survival of children with severe acute malnutrition (Trehan et al., 2013; Subramanian et al., 2014), however when the microbiome remained immature severe malnutrition re-occurred, suggesting that microbiome maturity may predict the long-term success of therapeutic food administration (ib.). Moreover, the enterotype appeared to contribute to the onset of severe malnutrition, an effect that could be reproduced by transplanting the human GI microbiota into gnotobiotic mice (Smith et al., 2013). Although current studies on human malnutrition are only observational, there are hopes that therapeutic remodeling of the GI microbiota may help eliminate malnutrition-associated mortality.

## Maintaining the GI microbiota during acute infections

During the host's response to pathogenic microorganisms initial anorexia can starve the pathogen while the host utilizes its own resources for resisting infection (Langhans, 2000). Anorexia could in principle also starve the microbiota; however, sophisticated preferential communication between the host and its microbiota was found to support the resident microbes during infection-induced stress. Lipopolysaccharides from the bacterial cell wall interact with and activate Toll-like receptors (TLRs) expressed on the membrane of intestinal macrophages. In mice TLR activation activated interleukin release and the fucosyltransferase 2 enzyme (Fut2) in the small intestine epithelial cells, resulting in fucosylation of certain membrane proteins (Pickard et al., 2014). Superficial L-fucose can be preferentially cleaved and utilized by the resident microbes (ib.). It was observed that *Fut2* polymorphisms may confer susceptibility to Crohn's Disease (Rausch et al., 2011) and its mutation both reduced microbiota diversity (Patwa et al., 2011; Wacklin et al., 2011) and increased sensitivity to *Candida albicans* infections in mice (Hurd and Domino, 2004). Contrary to initial reports, the bacteria-host interaction was found to be insufficient, albeit necessary, to trigger fucosylation in intestinal epithelial cells (Goto et al., 2014). Two additional mechanisms were found to contribute to fucosylation, the first being mediated by the type 3 innate lymphoid cells residing in the mucosal lining, which produce interleukin-22 in response to the resident microbiota and induce *Fut2* (*ib*). Reinforcing such mechanism, interleukin-22 also induces expression of the antibacterial RegIII-γ molecule (Satoh-Takayama et al., 2008; Sonnenberg et al., 2011; Tumanov et al., 2011). Finally, lymphotoxin α, also secreted by ILC3, could induce fucosylation independently of the microbiota (Patwa et al., 2011; Goto et al., 2014).

## Pathologies with altered GI microbiome

Because the GI microbiota interacts with the host immunity at multiple levels, dysbiosis may result in or be indicative of pathologies (Table 1). In fact, because of the complexity and reciprocity of the microbiota-host relationships it is debated whether the dysbiotic state may cause or be consequence of the pathology (Butto and Haller, 2016). Alterations of the composition of the GI microbiota may occur upon exposure to antibiotics, drugs, or radiation (Wallace et al., 2011; Nam et al., 2013; Keeney et al., 2014), stress or infections (Moloney et al., 2014), and nutritional changes (Griffin et al., 2017). Asthma and inflammatory bowel diseases (IBD) were both found to be directly affected by the interactions between the gut microbiota and host immunity.

**Table 1:** Diseases associated with GI microbiota abnormalities.

**Disease**	**Features**	**References**
Rheumatoid arthritis	Chronic, inflammatory auto-immune disorder displaying reduced *Bifidiobacteria* and *Bacteroidetes-Porphyromonas-Prevotella* group, *Bacteroidetes fragilis* subgroup, and *Eubacterium rectale-Clostridium coccoides* group, and increased *Lactobacillus*.	Wu et al., 2016
Inflammatory bowel disease	Dysbiotic inflammatory response to intestinal microbes. Increased numbers of innate immunity cells (neutrophils, macrophages, dendritic cells, and natural killer T cells) and adaptive immunity cells (B and T lymphocytes), which enact immune tolerance or defense against the intestinal microbiota.	Abraham and Cho, 2009; Halfvarson et al., 2016
Irritable bowel syndrome	Enrichment of Firmicutes and reduction of Bacteroidetes.	Krogius-Kurikka et al., 2009; Rajilić-Stojanović et al., 2011; Jeffery et al., 2012; Kennedy et al., 2014
Ulcerative colitis	Reduction of Bifidobacteria. Inflammation confined to the mucosa of the colon.	Abraham and Cho, 2009; Duranti et al., 2016
Crohn's disease	Reduction of Firmicutes and Bacteroidetes. Transmural inflammation.	Eckburg and Relman, 2007; Abraham and Cho, 2009
Ileal Crohn's disease	A form of Crohn's disease typified by decreased Paneth cell α-defensins, weakened antibacterial activity of the ileal mucosa, leading to bacterial composition changes in the microbiota.	Wehkamp et al., 2005
Type 1 diabetes	Auto-immunity against pancreatic β-cells (normally producing insulin) in genetically predisposed individuals. Defective development or alterations of the microbiota may result in dysregulated immunity with autoimmune β-cells destruction, and/or increased leakiness of the gut epithelial barrier. Decreased microbiome diversity.	Atkinson and Eisenbarth, 2001; Bosi et al., 2006; Vaarala et al., 2008; Atkinson et al., 2013; Dunne et al., 2014
Asthma	The airway microbiome is affected by outbreaks of *Chlamydophila pneumoniae* during the development of bronchitis and pneumonia. The GI microbiota is affected by the environmental exposure of microbes, especially early in life, which in turn affects the maturation of immune function to protect against allergic sensitization.	Hahn et al., 1991; Huang and Boushey, 2015
Obesity	Shift in the proportion of Firmicutes and Bacteroidetes with a significant increase of the former, leading to obesity in conjunction with poor diet.	Consortium THMP, 2012; John and Mullin, 2016
Obesity and gastric bypass	Significantly fewer *Firmicutes* compared to obese and healthy patients. Increase in *Gammaproteobacteria* in post-gastric-bypass patients.	Zhang et al., 2009
Cancer (various)	Carcinogenesis may develop in response to epithelial injury and inflammation from infectious agents, genetic mechanism, or pathogens (e.g., *Helicobacter pylori, Salmonella enterica, Borrelia burgdorferi, Chlamydia psittaci*).	Virchow, 1863; Balkwill and Mantovani, 2001; Grivennikov et al., 2010; Moore and Chang, 2010; Trinchieri, 2012; Schwabe and Jobin, 2013
Typhoid fever	Caused by infection of *Salmonella* species (spp.) *S. enterica* serovar Typhi (*S*. Typhi).	Rabsch et al., 2001; Crump and Mintz, 2010; Graham, 2010; Ahmer and Gunn, 2011
Food poisoning and foodborne pathogens	Opportunistic pathogens (e.g., *Campylobacter, Salmonella, E. coli, Shigella, Cronobacter, Listeria, Cryptosporidium*, MRSA, etc.) disrupt the equilibrium of the microbiome leading to dysbiosis, loss of host bacterial diversity and multiple diseases.	Brown et al., 2012; Shim, 2013; Carriere et al., 2015; Josephs-Spaulding et al., 2016
Malnutrition	Decrease or absence of species that either efficiently process food categories or produce vitamins may lead to reduced nutrient absorption and inflammation. *Enterobacteriaceae* overgrowth may result in epithelial damage, diarrhea, and reduced nutrient absorption.	Mohan et al., 2006; Lupp et al., 2007; Round and Mazmanian, 2009; Kane et al., 2015
*Clostridium difficile* Infection	A nosocomial pathogen, CDI is associated with epithelial inflammation and necrosis of the colon, diarrhea, pseudomembranous colitis and toxic megacolon. Antibiotic exposure may increase risk of re-infection.	Heinlen and Ballard, 2010; Khanna et al., 2016
Peptic ulcer disease	*Helicobacter pylori* are H_2_- receptor antagonists responsible for peptic ulcers, found in the stomach or duodenum. *H. pylori* was found to modify epithelial proliferation and apoptosis in gastric mucosa, reducing proliferation and increasing apoptosis *in vitro* in models of *H. pylori* infection.	Ding et al., 2008; Prabhu and Shivani, 2014
Chronic gastritis	*H. pylori* infection shown to increase epithelial cell turnover rate in gastric mucosa, with increased proliferation and apoptosis rates.	Wagner et al., 1997; Ahmed et al., 2000; Jang and Kim, 2000; Choi et al., 2003; Suzuki et al., 2004; Ernst et al., 2006; Ding et al., 2008
Gastric Mucosa-associated lymphoid tissue (MALT) tumors	Associated with *H. pylori* infections. Microbial virulence factors (*e.g., CagA*, and *VacA*) activate inflammatory processes and cell proliferation.	Fox and Wang, 2007; Francescone et al., 2014; Wang et al., 2014
Multiple sclerosis	Increased *Methanobrevibacter* (phylum Euryarchaeota) and *Akkermansia* (phylum Verrucomicrobia) and decreased *Butyricimoas* (phylum Bacteroidetes).	Jangi et al., 2015
Depression	*Bifidobacterium infantis*, normally found in GI of neonatal infants and in administered probiotic drugs may have antidepressant effects in psychobiological systems.	Desbonnet et al., 2010; Dinan et al., 2013; Evrensel and Ceylan, 2015
Anxiety	Oral administration of subclinical doses of *Campylobacter jejuni* in murine models induced anxiety-like behavior without stimulating immunity. *Lactobacillus* and *Bifidiobacterium* may function as anxiolytic influence in a murine model.	Sudo, 2006; Lyte et al., 1998; Bravo et al., 2011; Messaoudi et al., 2011; Barrett et al., 2012; Evrensel and Ceylan, 2015; Akkasheh et al., 2016; Schnorr and Bachner, 2016
Non alcoholic fatty liver disease	Reduced levels of Bacteroidaceae, *Bacteroides* and *Oscillospira*	Chierico et al., 2016
Diarrheal illness	Enteric infection of the jejunum caused by *Cyclospora cayetanensis*, a foodborne and waterborne parasite. Results in diarrhea, including what is referred to as *traveler's diarrhea*.	Ortega and Sanchez, 2010
Giardiasis	Infection of the protozoan *Giardia lamblia* transmitted through the consumption of contaminated drinking water inducing abdominal cramps, gas, nausea, and weight loss. In a murine model, *Giardia* colonization and proliferation affected commensal bacteria with decreased *Firmicutes and Melainabacteria*, and increased *Proteobacteria*.	Maloney et al., 2015; Barash et al., 2017

### Asthma

Asthma is a chronic disease that causes inflammation of the airways (NIH, 2014). In asthma, both low diversity and a numerically reduced gut microbiota eventually appears to induce allergy and lung hypersensitivity (Table 1, Ferreira et al., 2014). Common symptoms include wheezing, coughing, difficulty breathing, or shortness of breath with variable severity. It has been proposed that asthma prevalence may be increasing in Western countries because of lifestyle modifications that impoverish the microbiota, including excessive hygiene, liberal use of antibiotics and a high-fat diet (Strachan, 1989; Wills-Karp et al., 2001; Vercelli, 2009, 2010; Von Mutius and Vercelli, 2010; Allan and Devereux, 2011; Korpela et al., 2016). However, research is still developing about the long term effects of antibiotic use on the microbiota (WHO, 2014; Korpela et al., 2016). Epidemiological studies indicated that early life exposure to microbes helps preventing allergic diseases, leading to the “hygiene hypothesis” (Wills-Karp et al., 2001). This theory speculates that reduced microbial exposure due to aggressive cleaning, disinfection, urbanization, and impoverished diets may lead to altered microbiota and inadequate immune function (Ferreira et al., 2014). The findings of Weber et al. (2015) suggest that home or personal cleanliness may in fact have no impact on the development of asthma. Rather, a variety of microbial exposures may determine microbiota composition, possibly leading to the development of asthma in predisposed individuals (Ege et al., 2011, 2012; Weber et al., 2015). Asthmatic children were found to have prevalence of the pathogenic *C. difficile* and low abundance of non-pathogenic *Bifidobacterium* in their intestinal microbiota, which may reduce immune function in the airways (Kalliomäki et al., 2001a,b) and potentially contribute to disease chronicization.

Invariant natural killer T cells (iNKT cells) are part of innate immunity and mediate inflammation via interleukins IL-4 and IL-2. In a murine asthma model, germ-free mice were found to have higher counts of iNKT cells, compared to specific pathogen free mice (Ferreira et al., 2014). In contrast, germ-free mice colonized with normal mice microbiota appeared protected from the inflammatory effects of accumulated iNKT cells and from asthma (ib.). Interestingly, this was true only if gut colonization occurred in newborns, supporting the idea that early life microbial exposure may promote immune system proficiency and potentially offer disease protection later in life (Ferreira et al., 2014). Similarly, neonatal introduction of *Lactobacillus rhamnosus* GG, a probiotic strain of human origin, reduced allergic responses in the airways (ib.). *L. rhamnosus* was found to induce macrophages and to moderately activate the inflammasomes, cytoplasmic complexes responsible for the regulation of the pro-inflammatory response and induce macrophages (Martinon et al., 2009). Interestingly *L. rhamnosus* exposure was also found to cross-protect the mice from influenza A viral infection (Miettinen et al., 2012). Underscoring the complexity of host-microbe interactions, comparison of the global gene activation patterns elicited by the related *L. rhamnosus* GG and LC705 strains revealed that, despite their high degree of genetic relatedness, the two strains provoked distinct responses in the host and displayed different levels of gene activation with the induction of distinct gene sets (ib.).

### Inflammatory bowel disease

Inflammatory bowel disease (IBD) is an aggravating long-term inflammation of the intestine in which the commensal microbiota plays a key role in conjunction with individual genetic susceptibility (Elson et al., 2005; Obermeier et al., 2005; Schirbel and Fiocchi, 2010; Ferreira et al., 2014; Liu et al., 2015). IBD patients suffer intermittent severe bowel inflammation and displayed distinct composition of the gut microbiota, with decreased *Firmicutes* (Table 1, Walker et al., 2010; Ferreira et al., 2014). Abnormal GI microbiota has also been observed in subsets of patients affected by Crohn's disease and ulcerative colitis, two IBD variants (Park et al., 2017). Compared to healthy controls, the GI microbiota of IBD patients displayed fewer bacteria with anti-inflammatory properties and/or more bacteria with pro-inflammatory properties (Ott, 2004; Ferreira et al., 2014; Park et al., 2017). Inflammasome activation in the gut triggered a cascade culminating in release of interleukins IL-1β and IL-18, which in turn stimulated the inflammasome and amplified the inflammatory response (Miettinen et al., 2012). Corroborating a causal link between GI microbiota and IBD, the resident bacteria in the GI tract were found to prevent or alleviate IBD in murine models (Ferreira et al., 2014). Germ-free mice were prone to developing colitis and Crohn's disease, while gut re-colonization following gavage with healthy microbiota decreased inflammation (ib.). Early observations indicated that IBD patients displayed damaged intestinal barrier and increased intestinal permeability allowing microbe translocation and activation of the systemic immunity (Hollander et al., 1986). Translocation is a mechanism by which microbes can break through the gut barrier, enter the circulation and may reach other organs, unless targeted by innate and adaptive immunity. Revealing a degree of functional overlap, the gut microbiota was found to prevent translocation of the probiotic *E. coli* strain Nissle 1917 even if either the adaptive or innate immune system were to be defective (Gronbach et al., 2010). Conversely, in the absence of a GI microbiota the host's immunity could prevent microbial translocation (ib.). IBD susceptibility appeared to be linked to multiple defects affecting the gut barrier, Paneth cells, and innate immunity that result in decreased tolerance and increased immune response (Jostins et al., 2012; Buttó et al., 2015; Liu et al., 2015). Changes in resident microbiota composition following antibiotic treatment or other stressors (e.g., inflammation, Lupp et al., 2007) may favor the proliferation of pathogens, such as *C. difficile, Enterococcus, Salmonella*, and *Escherichia* species (Keeney and Finlay, 2011; Ng et al., 2013) causing further dysbiosis. These bacteria were found to efficiently utilize the nutrients in the gut environment and, at least in the case of *Salmonella*, be able to simultaneously implement adhesion mechanisms and bacteriotoxin production to, respectively, resist the host immunity and outcompete the resident microbiota (Raffatellu et al., 2009; Winter et al., 2010). IBD patients may also suffer the outgrowth of opportunistic pathogens or pathobionts, as a result of their unbalanced microbiota. Unlike the pathogens described above, these species are normally found within the human GI microbiota, yet they are kept under control. The imbalance of dysbiosis however, may favor their proliferation and the expression and/or proliferation of their virulent traits. Among these pathobionts were identified species of *Klebsiella, Proteus*, and *Escherichia* (Darfeuille-Michaud et al., 2004; Baumgart et al., 2007; Lupp et al., 2007; Walters et al., 2014; Buttó et al., 2015).

Finally, the high levels of inflammation characterizing IBD likely affect the microbiota as they do the host, inducing molecular and physiological changes. It was found that in response to harsh environments *E. coli*—which is found enriched in IBD patients (Table 1)—could up-regulate the *ibpA* and *ibpB* genes belonging to the stress response regulon, which are involved in cell division, signal transduction and intestinal adhesion (Zuckert, 2014; Tao et al., 2015). In the IL10^−/−^ IBD model mouse inoculated with a murine isolate of colitis-inducing *E. coli* (NC101), *ibpA* and *ibpB* were found to be activated, presumably to cope with the high amounts of reactive oxygen species induced by intestinal inflammation and—possibly—by the increased temperature caused by inflammation (Patwa et al., 2011). Surprisingly, this response impaired *E. coli* luminal growth and decreased survival after macrophage phagocytosis, which seemed counter-intuitive (ib.). While these observations may be partially limited due to the use of a monoassociated mouse and the focus on luminal bacteria, they indicate that the microbiota is rapidly and dynamically responding to the host conditions.

## Fecal transplantation

Recognizing that gut microbes are so integral to human health, the prospect of reconstituting a healthy microbiota in cases of dysbioses or disease becomes particularly attractive. Fecal microbiota transplantation (FMT) aims at reversing altered microbiota to their native state or one with healthy functionality. In FMT intestinal microbiota obtained from a healthy donor is processed, standardized and finally transplanted into the intestine of a recipient. FMT is being considered in alternative to antibiotic therapy to treat chronic *C. difficile* infections (CDI) that occur in dysbioses. The United States record over 500,000 annual cases of CDI, making it the leading cause of nosocomial diarrhea which, untreated, can result in lethal toxic megacolon (Bakken et al., 2011). Aggressive antibiotic therapies have produced chronic conditions with antibiotic resistant and hyper-virulent *C. difficile* strains overtaking a severely depleted GI microbiota (Cammarota et al., 2014; Khoruts, 2014). The current treatment for CDI is oral vancomycin administration, despite dwindling efficacies and a recorded 20–60% relapse within the first month following the completion of the course of vancomycin (Brandt et al., 2011). With limited options to eradicate *C. difficile* infections, FMT seems a viable alternative for restoring a healthy GI microbiota while avoiding the undesirable effects of antibiotics. CDI characteristic dysbiosis revealed dominating Gammaproteobacteria and Bacilli and rare Clostridia and Bacteroidetes, which were instead abundant in normal GI microbiota (Shankar et al., 2014). 16S rRNA profiling of the fecal bacteria following FMT showed apparent restoration of the microbial community and absence of *C. difficile* (Seekatz et al., 2014; Shankar et al., 2014). The decreased symptoms and clinical improvements remained stable for at least four months post procedure (Shankar et al., 2014), while FMT re-established a GI microbiota whose composition shifted from the diseased state and resembled more that of the donor (ib.). These landmark studies reported an impressive 90% success rate in reducing the debilitating CDI symptoms in a small patient cohort. The enthusiasm was tempered by finding much rarer successes in severe CDI (Zainah et al., 2014), albeit the procedure appeared safe and successful in appropriately chosen patients (Chapman et al., 2016). Although the transplanted microbiota appeared to generate a healthier microbiota taxonomy and could resolve the CDI symptoms (Bakken, 2009; van Nood et al., 2013; Zainah et al., 2014), many aspects of FMT demand further analyses and larger clinical studies. A better definition of the composition(s) of healthy gut microbiota and further understanding of how FMT affects the GI microbial community, its physiological interactions with the patient and how long the changed microbiota is maintained in the patients will all contribute to the further development of this procedure (Bakken, 2009; Brandt, 2013; Cammarota et al., 2014; Colman and Rubin, 2014; Khoruts and Weingarden, 2014; Moayyedi et al., 2014; Zainah et al., 2014; Dai et al., 2015).

## Concluding remarks

Convincing accumulating evidence shows that the human gut microbiota contributes to many aspects of human health via molecular pathways that we only begin to understand. The GI microbiota entertains deep mutualistic relationships and co-evolves with the human host, albeit at a much faster rate and demonstrates deep ecological links with the host which are being studied at the interface between Biology, Ecology, and Medicine. Experimental probing of the deep and reciprocal ties characterizing the microbiota-host relationship constitutes a formidable challenge, yet holds the promise to shed light on unknown aspects of human and microbial physiology and novel therapeutic possibilities in the manipulation of microbiota composition via antibiotics, probiotics, and microbial transplantation. Progress in microbiota studies and the recent discovery of the first new antibiotic in 30 years isolated from the pool of “un-culturable bacteria” (Ling et al., 2015) rekindle the hopes to find new therapeutics less prone to elicit microbial resistance. Because of the trophic relationships between host and gut microorganisms, dietary manipulation to supply appropriate nutrients favoring specific classes of beneficial microorganism may also aid future therapies for chronic pathologies displaying gut dysbiosis, such as asthma, IBD, and RA.

## Author contributions

This manuscript is the result of collaborative work during the Microbiology course (BIOL371) at Concordia University. MA, HAk, HAl, HBr, ABa, FB, VB, RB, ABr, MCa, TC, MCh, EK, AS contributed text on microbial adhesion and colonization mechanisms; SE, CE, NFe, NFl, TF, MGa, MGe, SG, NG, JB, TH contributed text on bacterial competition and foreign species invasion; JC, KC, FC, SD, IH, MI, SI, SK, PL, KL contributed text on the microbiota interactions with the host's immune system; DJ, AJ, MJ, MK contributed text on microbiome alterations and human pathologies; KK, CK, HK contributed text on the gut brain axis; SL, JL, YM, PM, ML, EN, IN, TNg, RN, MO'D, MOu, VS, TNa, TU contributed text on fecal transplant; YM, PM, TU, VS also contributed text on legal implications of the fecal transplants that allowed the development of the review in its current state, albeit it is not represented in the current manuscript; SP, NP, EP, JM contributed text on the emerging roles for the gut microbiome and new perspectives; JP, AP, CP, VP, HR, RR, JR, DR contributed text on the relationships between diet and microbiome; BR, SSa, MS, SSe, LS, AS, DS, contributed text on the efficacy of supplementation and persistence of probiotic strains; MVa, MVi contributed text on microbiota and fasting; PV, LW, CZ, PdS, MW contributed text on commensal microorganisms and cancer; AA, TA, LA, IB contributed text on anti-microbial peptides; HBe, MDa, MDe, TD, SDi, SDo, PD, FD, LD contributed text on antibiotics and antibiotic resistance that was excluded in part from the text of the revised document, yet contributed to the article in its final format. In addition, SSe and BR collaborated to fuse all individual contributions into a cohesive manuscript; CG conceived, organized, and edited the manuscript. All authors read and approved the final manuscript.

### Conflict of interest statement

The authors declare that the research was conducted in the absence of any commercial or financial relationships that could be construed as a potential conflict of interest.
